# Dominant Orbitofrontal Pial Supply in Anterior Cranial Fossa Dural Arteriovenous Fistula: Angiographic Differentiation from Mixed Pial-Dural Arteriovenous Malformation and Anatomy-Based Treatment Selection

**DOI:** 10.3390/brainsci16050534

**Published:** 2026-05-19

**Authors:** Kosei Goto, Nobuo Kutsuna, Takuto Nishihara, Kotaro Makita

**Affiliations:** 1 Department of Neurosurgery, Fukujukai Adachi Tobu Hospital, Tokyo 121-0816, Japan; k.goto@fukujukaigr.or.jp (K.G.);; 2 Department of Stress and Invasiveness Control, Toho University School of Medicine, Tokyo 153-8515, Japan

**Keywords:** cerebrovascular disease, dural arteriovenous fistula, anterior cranial fossa, pial arterial supply, orbitofrontal artery, mixed pial-dural arteriovenous malformation, angiography, treatment selection, mechanical thrombectomy, microsurgery

## Abstract

**Highlights:**

**What are the main findings?**

- A high-grade anterior cranial fossa dural arteriovenous fistula with dominant orbitofrontal pial supply was recognized during mechanical thrombectomy for cardioembolic stroke.
- Angiographic assessment supported anterior cranial fossa dural arteriovenous fistula rather than mixed pial-dural arteriovenous malformation, and microsurgical draining-vein disconnection achieved complete obliteration.

**What are the implications of the main findings?**

- In pial-feeder-dominant anterior cranial base shunts, differentiation from mixed pial-dural arteriovenous malformation and careful evaluation of endovascular feasibility are central to treatment selection.
- When pial transarterial access is unsafe and complete transvenous closure is uncertain, microsurgical disconnection remains a reliable curative option.

**Abstract:**

Background: Anterior cranial fossa dural arteriovenous fistulas (ACF DAVFs) usually receive ethmoidal dural supply. Pial arterial supply has been described in intracranial DAVFs, including ACF DAVFs, but a dominant orbitofrontal pial feeder can create diagnostic overlap with mixed pial-dural arteriovenous malformation and make endovascular treatment hazardous. Case Presentation: A 75-year-old man with atrial fibrillation presented with right middle cerebral artery occlusion and underwent intravenous thrombolysis followed by mechanical thrombectomy. During right internal carotid angiography, transient arterial-phase opacification of a contralateral frontal draining vein through the anterior communicating artery prompted post-recanalization angiography. A high-grade left ACF DAVF was diagnosed, with dominant supply from the left orbitofrontal artery, minor anterior ethmoidal supply, two venous drainage routes, cortical venous reflux, and a varix. Although the DAVF was incidental to the ischemic presentation, it was considered to require treatment because of high-risk angioarchitecture, including Borden type III/Cognard type IV drainage, cortical venous reflux, and venous ectasia. No intraparenchymal nidus or normal venous-phase use of the refluxing veins was identified. Because pial transarterial access and complete transvenous closure were considered unsafe or uncertain, microsurgical draining-vein disconnection was performed. Postoperative angiography confirmed complete obliteration. Conclusions: In this case, microsurgical disconnection achieved angiographic cure, and the patient was transferred for rehabilitation with a modified Rankin Scale score of 1. The central diagnostic and therapeutic issue in pial-feeder-dominant ACF DAVF is not rarity alone, but angiographic differentiation from mixed pial-dural arteriovenous malformation and assessment of whether the shunt can be closed without compromising normal pial arteries or venous outflow. The thrombectomy angiogram provided the route to diagnosis, whereas pial arterial dominance and divided venous drainage determined the curative strategy.

## 1. Introduction

Anterior cranial fossa dural arteriovenous fistulas (ACF DAVFs) are uncommon intracranial DAVFs, but many drain directly into cortical veins because no large venous sinus lies immediately at the anterior cranial base. This angioarchitecture is associated with cortical venous reflux, venous varices, and hemorrhagic presentation [1,2,3,4]. The usual arterial supply is from the anterior ethmoidal artery, arising from the ophthalmic artery [5]. In contrast, pial arterial supply from the anterior cerebral artery has been reported but is uncommon, and it becomes especially relevant when it is the dominant supply [6]. When a pial feeder is the main supply, the lesion may be difficult to distinguish from a mixed pial-dural arteriovenous malformation (AVM), and the feasibility of safe endovascular access becomes a separate therapeutic issue [7,8].

Mechanical thrombectomy has become a central treatment for acute ischemic stroke caused by large-vessel occlusion [9]. In a time-pressured stroke procedure, an unexpected angiographic finding outside the culprit territory may be dismissed as incidental or artifactual. In the present case, however, the stroke angiogram was not the main novelty but the route by which a high-risk shunt was recognized. The more durable lesson was the angioarchitectural problem that followed: a dural fistula at the anterior cranial base was supplied mainly by an orbitofrontal pial artery and drained through two venous routes, creating diagnostic overlap with mixed pial-dural AVM and uncertainty regarding safe endovascular closure.

We report a high-grade ACF DAVF with dominant orbitofrontal pial supply that was recognized during acute reperfusion therapy for embolic middle cerebral artery occlusion. The case is not presented simply as a rare feeding pattern, but as an anatomy-based diagnostic and management problem. We emphasize how the dominance of the orbitofrontal pial feeder, absence of a demonstrable parenchymal nidus, divided venous outflow, and the risks of transarterial or transvenous embolization shaped definitive treatment selection.

## 2. Case Presentation

### 2.1. Acute Stroke Presentation and Thrombectomy

**Figure 1:**
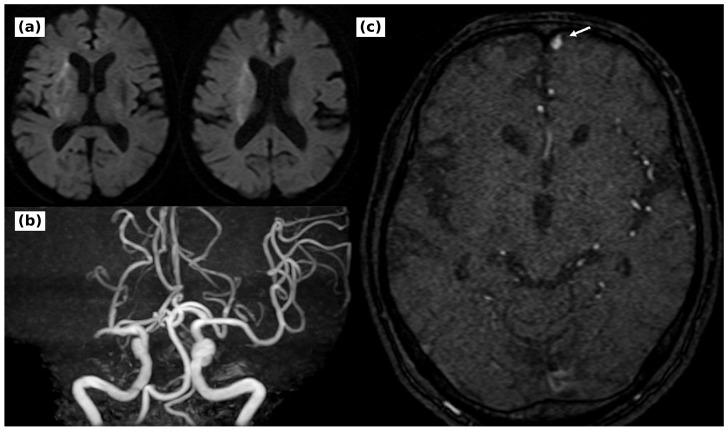
Initial magnetic resonance imaging and retrospective review of time-of-flight magnetic resonance angiography. (**a**) Diffusion-weighted imaging showed faint hyperintensity in the right basal ganglia and corona radiata; the diffusion-weighted imaging-Alberta Stroke Program Early Computed Tomography Score was 9. (**b**) Magnetic resonance angiography suggested occlusion of the right M1 segment. (**c**) Retrospective review of the pretreatment time-of-flight source image demonstrated an abnormal vessel in the left frontal region (white arrow).

A 75-year-old man presented with sudden dysarthria and left hemiparesis. He had atrial fibrillation and had taken warfarin until the day of onset; the international normalized ratio was 1.3 on arrival. The National Institutes of Health Stroke Scale score was 18. Magnetic resonance angiography suggested right M1 occlusion, and diffusion-weighted imaging showed faint hyperintensity in the right basal ganglia and corona radiata, with a diffusion-weighted imaging-Alberta Stroke Program Early Computed Tomography Score of 9 (Figure 1). Cardioembolic occlusion of the right middle cerebral artery was suspected, and intravenous recombinant tissue plasminogen activator was followed by mechanical thrombectomy.

**Figure 2:**
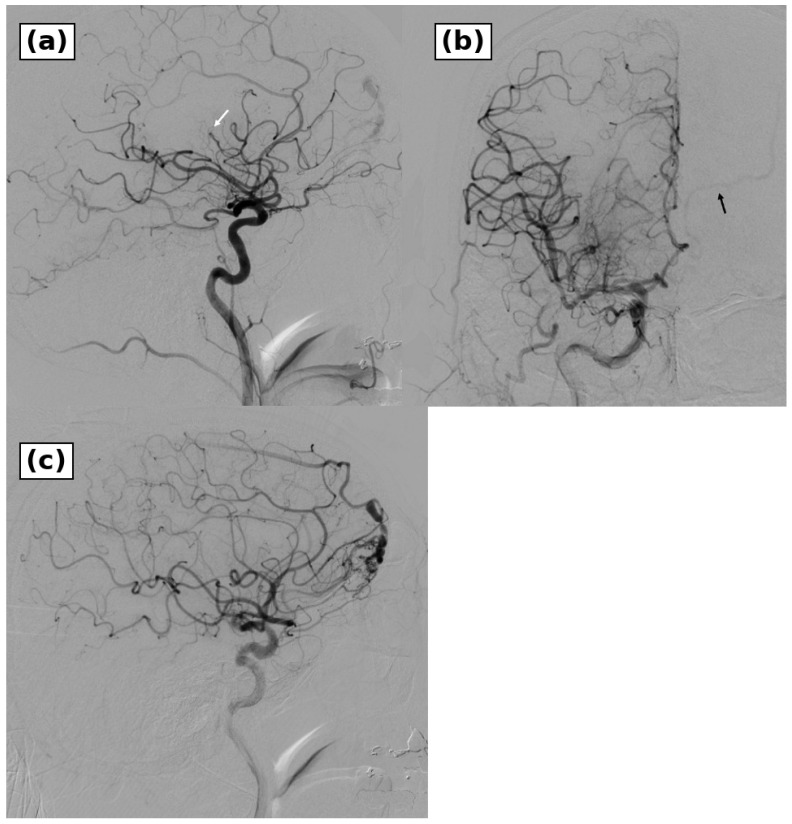
Right internal carotid artery angiography during mechanical thrombectomy. (**a**) Lateral right internal carotid artery angiography showed that the right M1 segment had already recanalized, whereas a central branch of the middle cerebral artery remained occluded (white arrow). (**b**) In the arterial phase of the right internal carotid artery injection, cross-filling through the anterior communicating artery transiently opacified an abnormal venous channel in the contralateral frontal region (black arrow). (**c**) Recanalization was achieved by aspiration through a microcatheter, after which targeted angiography was performed to evaluate the abnormal venous filling.

Initial right internal carotid angiography showed that the M1 trunk had already recanalized, whereas a central branch of the middle cerebral artery remained occluded (Figure 2). Because left hemiparesis persisted, aspiration through a microcatheter was performed, and recanalization was achieved. The left hemiparesis improved promptly after reperfusion. During the arterial phase of right internal carotid injection, however, cross-filling through the anterior communicating artery transiently opacified a contralateral abnormal venous channel from the left anterior cranial base to the left ascending frontal and frontal bridging veins (Figure 2). This early venous filling was remote from the culprit ischemic territory and was not explained by normal venous drainage. Additional angiography was therefore performed after completion of thrombectomy.

### 2.2. Angioarchitectural Diagnosis and Treatment Selection

**Figure 3:**
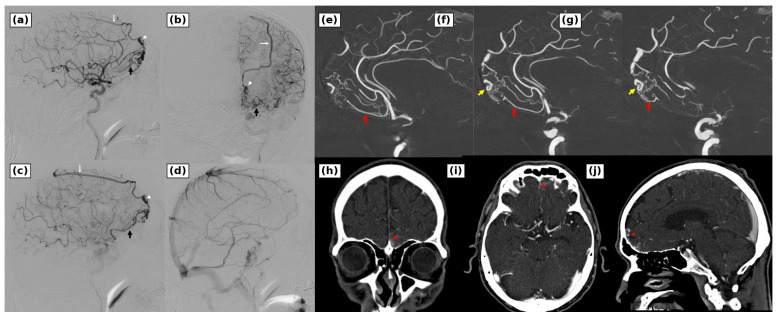
Diagnostic and surgical planning images of the anterior cranial fossa dural arteriovenous fistula. (**a**,**b**) Diagnostic left internal carotid artery angiography demonstrated a high-grade fistula at the left anterolateral cribriform plate, supplied predominantly by the left orbitofrontal artery with minor contributions from the right orbitofrontal artery and the left anterior ethmoidal artery. The fistula drained into the left ascending frontal vein and frontal bridging vein (white arrow) with a venous varix (white arrowheads), and also into the posterior fronto-orbital venous route (black arrow). (**c**,**d**) Later angiographic images demonstrated the lesion-related venous outflow and preserved normal venous drainage. (**e**–**g**) Three-dimensional rotational angiography delineated the steep orbitofrontal feeder arising from the anterior cerebral artery (red arrow), the shunt point (yellow arrow), cortical venous reflux, the venous varix, and the posterior fronto-orbital venous outflow toward the lateral cavernous sinus and pterygoid plexus. (**h**–**j**) Computed tomography angiography localized the fistulous point to the left lateral cribriform plate (red arrowheads) and demonstrated its relationship to the frontal sinus.

Left internal carotid angiography demonstrated a non-sinus-type ACF DAVF with a shunt point localized to the left anterolateral cribriform plate (Figure 3). The lesion was classified as Borden type III and Cognard type IV. The dominant feeder was the left orbitofrontal artery. The right orbitofrontal artery and the left anterior ethmoidal artery made minor contributions, and bilateral external carotid angiography showed no definite external carotid supply. The fistula drained through two routes: from the left ascending frontal vein to the frontal bridging vein and superior sagittal sinus, and from the left frontobasal bridging vein to the posterior fronto-orbital vein, lateral cavernous sinus, and pterygoid plexus. A venous varix was present on the superior sagittal sinus route.

The refluxing veins were not opacified during the normal venous phase and were interpreted as lesion-related draining veins rather than physiologic cortical drainage. Deep venous drainage was preserved, and no pseudophlebitic pattern was seen. No intraparenchymal nidus or abnormal vascular tangle continuous with the brain parenchyma was identified. Retrospective review of the pretreatment time-of-flight magnetic resonance angiography source images showed an abnormal vessel in the left frontal region (Figure 1). Three-dimensional rotational angiography showed that the left orbitofrontal feeder branched sharply from the anterior cerebral artery and approached the shunt point from below. Computed tomography angiography localized the shunt to the left lateral cribriform plate and defined its relationship to the frontal sinus (Figure 3).

Because the lesion had cortical venous reflux and a venous varix, definitive treatment was planned during the same hospitalization. Edoxaban 30 mg/day was started on day 2 for secondary prevention of cardioembolic stroke after neurological improvement and limited infarct volume had been confirmed. Treatment of the DAVF was scheduled for day 16, balancing the hemorrhagic risk of the fistula against perioperative safety after acute ischemic stroke. Edoxaban was temporarily interrupted for intracranial surgery and resumed on postoperative day 3 after postoperative imaging confirmed no hemorrhagic complication; the timing was individualized according to infarct volume and operative bleeding risk. Transarterial embolization was not chosen as the first-line strategy because the main access route was a pial feeder from the anterior cerebral artery, the orbitofrontal branch arose at a sharp angle, and safe distal catheterization to the shunt point was unlikely. In addition, embolization from this route could have compromised normal pial branches through reflux or wedge-related distal occlusion. Transvenous embolization was also considered uncertain because the two venous outflow routes required stable access to the shunt pouch and complete closure of both the superior sagittal sinus and cavernous sinus directions. If venous occlusion had been incomplete, altered outflow through the posterior fronto-orbital route could have left or created a dangerous deep venous reflux pattern. For these reasons, microsurgical draining-vein disconnection was selected.

### 2.3. Microsurgical Disconnection and Outcome

**Figure 4:**
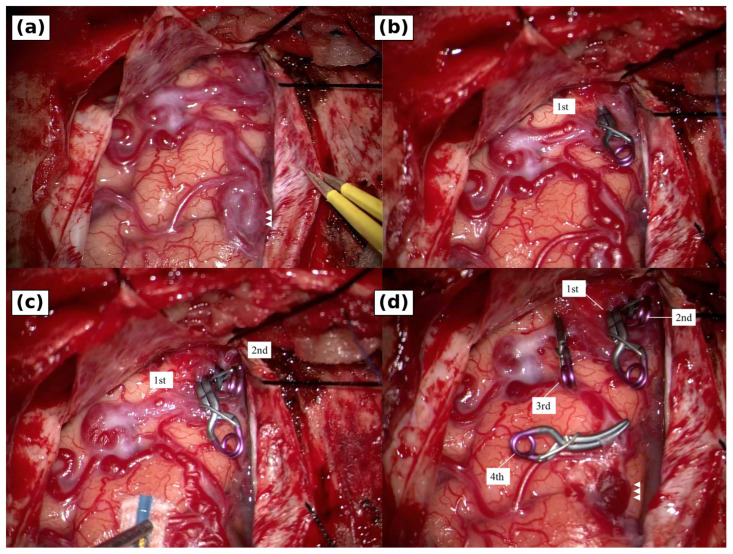
Intraoperative findings during microsurgical draining-vein disconnection. (**a**) After dural opening, the arterialized draining vein and venous varix (white arrowheads) were identified on the left anterior skull base. (**b**) The first clip was placed at the presumed shunt-to-draining-vein transition; however, indocyanine green videoangiography showed persistent fluorescence in the drainer, indicating residual outflow. (**c**) The second clip was placed on the distal draining vein toward the superior sagittal sinus pathway. (**d**) The third clip was placed before the posterior fronto-orbital venous outflow, and the fourth clip was added near the varix as a safeguard against residual channels. The final view showed collapse of the draining system. Labels indicate the order of clip placement.

A bifrontal craniotomy was performed through a coronal skin incision. The frontal sinus was opened, and the mucosa was trimmed and closed with 6-0 nylon sutures. After Y-shaped dural opening and anterior skull base exposure, an arterialized draining vein and venous varix were identified at the lateral left cribriform plate (Figure 4). A first clip was placed at the presumed transition from the shunt point to the draining vein. The vein and varix changed from red to blue, but indocyanine green videoangiography showed persistent fluorescence in the draining system, indicating residual outflow. A second clip was added on the route toward the superior sagittal sinus, and a third clip was placed before the posterior fronto-orbital venous outflow to avoid leaving the deep drainage route. A fourth clip was added near the varix as a safeguard against residual channels. The draining system then collapsed.

**Figure 5:**
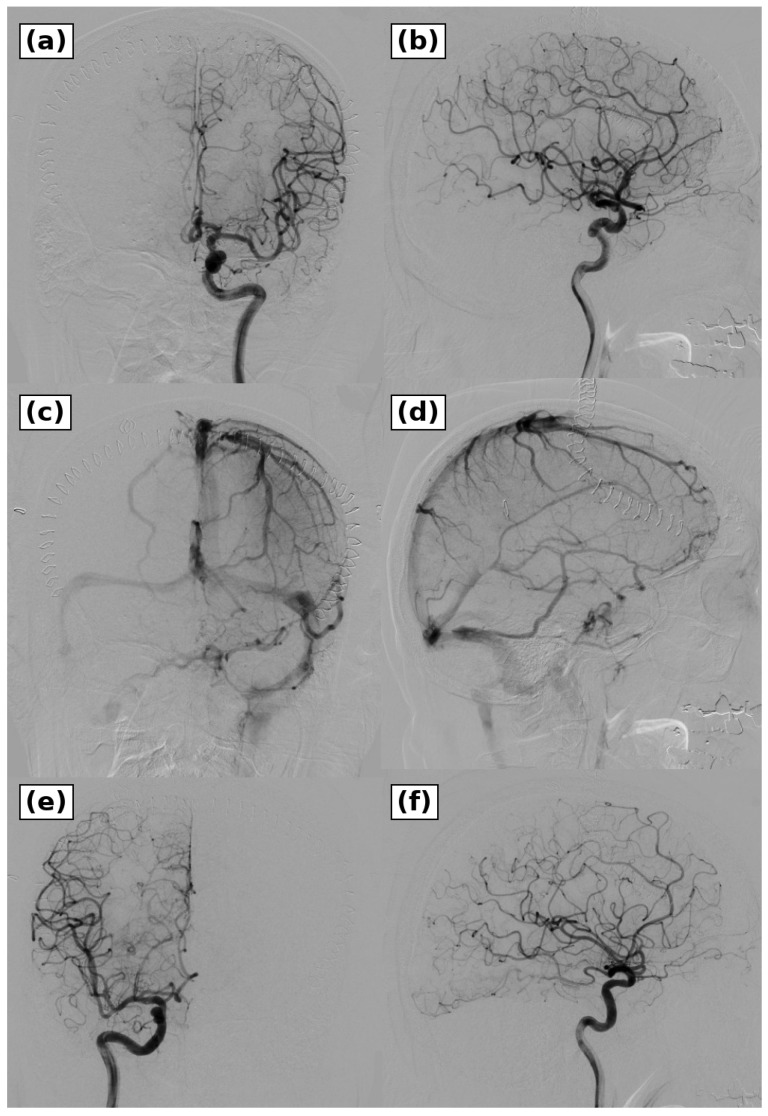
Postoperative angiography after microsurgical treatment. (**a**,**b**) Left internal carotid artery angiography obtained on postoperative day 3 showed complete obliteration of the fistula and absence of residual abnormal venous reflux in the arterial phase. (**c**,**d**) Left internal carotid artery angiography in the venous phase showed preserved normal superficial and deep venous drainage. (**e**,**f**) Right internal carotid artery angiography in the arterial phase confirmed absence of residual cross-filling or recurrent shunt. These findings confirmed complete obliteration of the fistula.

Postoperative computed tomography showed no new infarction or intracranial hemorrhage. The patient was alert, had no paresis, and could walk. Bilateral internal carotid angiography on postoperative day 3 confirmed complete obliteration of the fistula, no residual abnormal venous reflux, and preserved normal superficial and deep venous drainage (Figure 5). He was transferred for rehabilitation with a modified Rankin Scale score of 1. The ischemic event was attributed to cardioembolism from atrial fibrillation, and the ACF DAVF was considered an incidental but high-risk comorbid cerebrovascular lesion detected during reperfusion therapy.

## 3. Discussion

The principal lesson of this case is that dominant pial supply changed both the diagnosis and the treatment plan. Anterior cranial fossa dural arteriovenous fistulas (ACF DAVFs) are usually supplied by ethmoidal branches of the ophthalmic artery [5]. Pial arterial supply in ACF DAVFs has been described previously [6], so the educational value of the present case should not be based on rarity alone. The distinctive feature was the combination of a dominant orbitofrontal pial feeder, a dural shunt point at the lateral cribriform plate, divided venous outflow, and diagnostic overlap with mixed pial-dural arteriovenous malformation (AVM) [7,8]. This distinction matters because disconnection of only the draining vein in a mixed pial-dural AVM may leave a residual parenchymal nidus and carry a postoperative hemorrhage risk [7]. In the present case, several findings supported ACF DAVF rather than mixed pial-dural AVM: a shunt localized to the lateral cribriform plate, minor anterior ethmoidal supply, absence of an intraparenchymal nidus on selective angiography and three-dimensional rotational angiography, lack of physiologic use of the refluxing veins in the normal venous phase, and complete disappearance on bilateral carotid angiography after draining-vein disconnection. Nevertheless, the diagnosis remained angiographic rather than pathological, because no nidus-bearing specimen was obtained. A very small, compressed, or thrombosed parenchymal component cannot be excluded with the same certainty as pathological examination. We therefore interpreted the lesion based on convergent angioarchitectural findings rather than on a single imaging sign. Pial arterial supply is also an anatomic risk factor during treatment; Hetts et al. reported more treatment-related ischemic complications in intracranial DAVFs with pial arterial supply [10]. This risk was central to our decision not to embolize through the orbitofrontal feeder.

Treatment selection should be separated from diagnostic labeling. Microsurgical interruption of the draining vein has long been a reliable curative treatment for ethmoidal or anterior cranial base DAVFs [11,12,13,14,15]. At the same time, endovascular therapy for ACF DAVFs has improved, and recent series have reported successful transarterial embolization through ethmoidal or ophthalmic routes, transvenous embolization, or combined strategies [12,13,16,17,18,19]. In recent endovascular series, immediate or follow-up complete occlusion has ranged from approximately 69% to 91%, with procedure-related complications reported in approximately 0% to 9% of patients or procedures [16,17,18,19]. By comparison, a recent systematic review and meta-analysis of ethmoidal DAVFs reported higher complete obliteration after microsurgery than after endovascular treatment (89% vs. 70%) and similar procedure-related complication rates (10% vs. 13%) [20]. These aggregate data support an increasingly active role for endovascular treatment, but they do not directly settle management of an orbitofrontal pial-feeder-dominant lesion.

In this patient, the dominant feeder was a sharply angulated pial branch from the anterior cerebral artery, there was no safer external carotid route, and the venous side divided into superior sagittal sinus and cavernous sinus pathways. A single clip did not eliminate all outflow intraoperatively, and sequential clipping was needed to close both directions. This operative finding supports the preoperative concern that incomplete endovascular occlusion could have left residual or redirected venous reflux. Thus, microsurgical disconnection was chosen not because surgery was considered universally preferable, but because the patient-specific arterial and venous anatomy made pial transarterial access unsafe and complete transvenous closure uncertain.

The clinical context is relevant because the ACF DAVF was recognized during angiography for acute ischemic stroke, not during a hemorrhagic work-up. ACF DAVFs are rare; in the Japanese Registry of Neuroendovascular Therapy 2, anterior cranial fossa lesions accounted for 13 of 1075 intracranial DAVFs treated endovascularly, and ACF location, cortical venous reflux, and venous varix were associated with hemorrhagic presentation [1]. The untreated natural history of DAVFs with cortical venous reflux is also unfavorable, especially when venous ectasia is present [4]. Although the present DAVF was not responsible for the ischemic symptoms, it was a Borden type III/Cognard type IV lesion with a varix. Observation alone was therefore considered inappropriate, particularly because anticoagulation was required for atrial fibrillation after the index stroke.

Retrospective review showed that an abnormal left frontal vessel was visible on the pretreatment time-of-flight magnetic resonance angiography source image. Enlarged cortical veins on magnetic resonance imaging can contribute to early diagnosis of ACF DAVFs [21]. Prior reports have described intravenous thrombolysis or mechanical thrombectomy in patients with a coexisting DAVF, and de novo DAVF after mechanical thrombectomy [22,23,24]. The present case adds a practical point: arterial-phase venous filling during thrombectomy led to recognition of an ACF DAVF whose dominant orbitofrontal pial supply and divided venous drainage made the distinction from mixed pial-dural AVM and the choice of curative strategy nontrivial. During hyperacute stroke care, reducing door-to-puncture time takes priority, and there may be little time for additional T2-weighted imaging or detailed source-image review. When arterial-phase venous filling appears outside the culprit territory during thrombectomy, especially through cross-filling, it should not be dismissed as routine venous-phase filling. After recanalization has been achieved, targeted additional angiography can define a coexisting high-risk vascular lesion without delaying the initial reperfusion procedure.

This report has several limitations. It is a single case; T2-weighted imaging was not obtained during the initial emergency magnetic resonance protocol, intraoperative digital subtraction angiography was not used, and no pathological specimen was available. For this reason, the exclusion of mixed pial-dural AVM should be regarded as angiographic, although the preoperative angioarchitecture, intraoperative appearance, and complete postoperative angiographic obliteration strongly supported ACF DAVF. The timing of surgery after ischemic stroke and the perioperative anticoagulation strategy cannot be generalized from one patient. The value of the case lies in the angioarchitectural recognition of a pial-feeder-dominant ACF DAVF, the differentiation from mixed pial-dural AVM, and the individualized selection of curative treatment after endovascular feasibility had been assessed.

## 4. Conclusions

A high-grade ACF DAVF with dominant orbitofrontal pial supply was recognized during acute reperfusion therapy for cardioembolic stroke. The main issue was not simply incidental detection during thrombectomy or the rarity of a pial feeder, but the angioarchitecture of the fistula: a dominant orbitofrontal pial feeder, no demonstrable parenchymal nidus, two venous drainage routes, and a varix. In pial-feeder-dominant anterior cranial base lesions, the shunt point, presence or absence of a parenchymal nidus, venous outflow routes, and relationship to normal venous return should be assessed carefully. In selected cases with unsafe pial transarterial access and complex venous outflow, microsurgical draining-vein disconnection remains a curative option after endovascular feasibility has been considered.

## Data Availability

The data presented in this case report are not publicly available because they contain patient-level clinical and imaging information. Additional anonymized information may be available from the corresponding author upon reasonable request, subject to the documented publication consent and institutional policy.
